# Sound Recognition Method of Coal Mine Gas and Coal Dust Explosion Based on GoogLeNet

**DOI:** 10.3390/e25030412

**Published:** 2023-02-24

**Authors:** Xingchen Yu, Xiaowei Li

**Affiliations:** School of Mechanical Electronic and Information Engineering, China University of Mining and Technology (Beijing), Beijing 100083, China

**Keywords:** gas and coal dust explosion, sound recognition, image recognition, continuous wavelet analysis, GoogLeNet

## Abstract

To solve the problems of backward means of coal mine gas and coal dust explosion monitoring, late reporting, and low leakage rate, a sound recognition method of coal mine gas and coal dust explosion based on GoogLeNet was proposed. After installing mining pickups in key monitoring areas of coal mines to collect the sounds of the working equipment and the environment, the collected sound was analyzed by continuous wavelet to obtain its scale coefficient map. This was then imported into GoogLeNet to obtain the recognition model of coal mine gas and coal dust explosions. The test sound was obtained by continuous wavelet analysis to obtain the scale coefficient map, brought into the completed training recognition model to obtain the sound signal class, and verified by experiment. Firstly, the scale coefficient map extracted from the sound signal by continuous wavelet analysis showed that the similarity between the subjective and objective indicators of the wavelet coefficient maps of the gas explosion sound and coal dust explosion sound was higher, but the difference between these and the rest of the coal mine sounds was clearer, helping to effectively distinguish gas and coal dust explosion sounds from other sounds. Secondly, the experimental results of GoogLeNet parameters can be obtained. When the dropout parameter is 0.5 and the initial learning rate is 0.001, the recognition effect of the model established by GoogLeNet was optimal. According to the selected parameters, the training loss, testing loss, training recognition rate, and testing recognition rate of the model are all in line with expectations. Finally, the experimental recognition results show that the recognition rate of the proposed method is 97.38%, the recall rate is 86.1%, and the accuracy rate is 100% for the case of a 9:1 ratio of test data to training data, and the overall recognition effect of the proposed GoogLeNet is significantly better than that of vgg and Alexnet, which can effectively solve the problem of under-sampling of coal mine gas and coal dust explosion sounds and can meet the need for the intelligent recognition of coal mine gas and dust explosions.

## 1. Introduction

Gas explosions, coal dust explosions, and gas and coal dust explosions (hereinafter referred to as gas and coal dust explosions) in coal mines are serious accidents [1] that cause a large number of casualties and economic losses. Therefore, there has been ongoing research and scholarship on this topic of study. Such research is mainly focused on the detonation conditions [2,3], explosion suppression materials [4,5], unsafe behavior [6], and other aspects.

In order to properly recognize imminent gas and coal dust explosions and raise the alarm, Refs. [7,8] studied the characteristics of explosion sounds in the time and frequency domains, as they are different from other sounds. It was proposed to detect coal mine gas and coal dust explosions through sound intelligence analysis and analysis of characteristic parameters such as sound frequency, amplitude, and short-time energy. The study in [9] characterized the sound signal by the energy–entropy ratio obtained from the dual-tree complex wavelet transform and classifies the sound using an Extreme Learning Machine (ELM) to identify gas and coal dust explosion sounds. To further improve the accuracy of coal mine gas and coal dust explosion identification, it is necessary to conduct in-depth research on the sound identification of coal mine gas and coal dust explosions. The study in [10] used Complementary Ensemble Empirical Mode Decomposition (CEEMD) to decompose the sound signal, obtain the sample entropy of the modal components, constitute the feature quantity of the sound signal, and then input the feature quantity to a Support Vector Machine (SVM) for the recognition and classification of the sounds of coal dust explosions.

Previous sound recognition research mainly selects suitable sound features and a recognition classifier to complete the recognition classification work. With the changing scenarios of sound recognition, the combination of sound recognition and image recognition has become a new means of sound recognition. Many scholars have done in-depth research on this aspect. The studies in [11,12] analyzed the characteristics of the lung sound spectrogram and proposed a wheezing sound recognition method based on the lung sound spectrogram, which provided reasonable hints and visual clues for pathological lung sound recognition. In [13], a sound event recognition method based on spectrogram texture features was proposed for the recognition of sound events in various environments. In [14], for the sound scene recognition problem in complex sound scene recognition tasks, a sound spectrogram extraction neural network is proposed to replace the traditional Meier inverse spectrum extraction process, and the sound spectrogram is automatically adapted to the sound scene dataset by training the network. With the continuous development of deep learning technology, GoogLeNet has been widely used in the field of pattern recognition. In [15], an accurate classification method based on wavelet decomposition of 1D-GoogLeNet was proposed to achieve the intelligent classification of cardiac arrhythmias.

**Figure 1:**
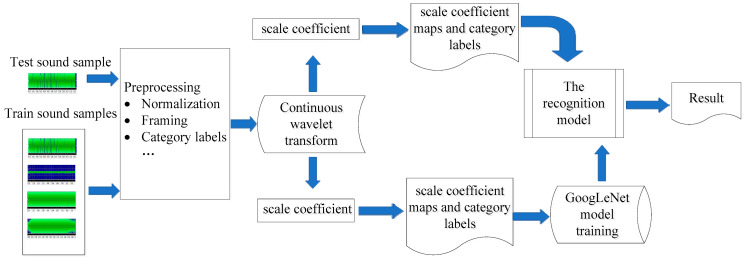
Working principle diagram.

Inspired by the research of the above researchers, a sound recognition method of coal mine gas and coal dust explosions based on GoogLeNet was proposed by analyzing its coefficient map through wavelet transform after analyzing the sound signals collected in the field from underground coal mines, as shown in Figure 1. Firstly, mining pickups are installed in key monitoring areas, such as rock faces being mined, the comprehensive excavation working face, the top of the roadway, and the roadway gang in underground coal mines, to collect the sound in the monitoring areas in real time; secondly, the collected sound signals are pre-processed by pre-emphasis, windowing, and framing, and then the coefficients are obtained by continuous wavelet transform to obtain coefficient maps, which are input to GoogLeNet for training to build coal mine gas and coal dust explosion sound recognition models for recognizing coal mine gas and coal dust explosions; finally, the test sound signals are also analyzed by continuous wavelet to obtain their coefficient maps, which are input to the trained recognition model to complete the sound recognition classification.

## 2. Preprocessing

Since the collected underground sound signal samples are long, the features cannot be extracted directly. To facilitate signal analysis and feature extraction, the sound signal needs to be pre-processed, and the pre-processing steps include normalization, framing, windowing, and adding category labels.

### 2.1. Normalization

This paper uses the mean value method to normalize the sound signal to the mean value.

### 2.2. Framing

The sound signal is characterized by short-time smoothness. Therefore, this paper uses the Hamming window to split the sound signal into frames, and the non-overlapping part of the frames is the frameshift. This system uses a Hamming window with a frame length of 20 ms and a frameshift of 10 ms. This kind of framing has two effects: (1) it can reduce the interference of silent audio; (2) it can reduce the difficulty of audio post-processing, optimize the algorithm process, simplify the calculation, and improve the computing speed and recognition efficiency of the recognition system.

### 2.3. Category Labels

To train the recognition model, this paper assigns different category labels to different sounds.

## 3. Continuous Wavelet Transform

The sound signal is a one-dimensional time series with low dimensionality of information features, and the sound signal collected from underground coal mines contains unnecessary high-frequency noise information. To increase the data dimensionality and denoising, this paper uses multi-resolution continuous wavelet transform to decompose the data, which both obtains the subsignals of each frequency band and increases the signal dimensionality, so that the model proposed in this paper achieves better recognition results.

The continuous wavelet analysis mainly decomposes the original signal into subsignals of different frequency bands by the scale function $\emptyset_{0,k}\left(m\right)$ and the wavelet function $\psi_{j,k}\left(m\right)\;$[16,17]. The wavelet approximation coefficients $a_{0}\left(k\right)$ and detail coefficients $d_{j}\left(k\right)$ of the assumed signal s(t) can be expressed as

(1) $$a_{0}\left(k\right)=\frac{1}{\sqrt{M}}\overset{M}{\underset{m=1}{\sum }}s\left(m\right)\emptyset_{0,k}\left(m\right)$$

(2) $$d_{j}\left(k\right)=\frac{1}{\sqrt{M}}\overset{M}{\underset{m=1}{\sum }}s\left(m\right)\psi_{j,k}\left(m\right)$$

where *j* is the scaling of the subsignal in the frequency domain, and *k* is the frequency shift of the subsignal in the frequency domain.

The original signal can be reconstructed according to the approximation and detail coefficients, as shown in Equation (3):

(3) $$s\left(t\right)=a_{0}\left(k\right)+d_{j}\left(k\right)$$

To preserve the frequency characteristics of the sound signal, the Morse wavelet [18,19] with better time-frequency resolution is chosen, which is a fully resolved wavelet and does not have negative frequency leakage. The coefficients obtained by Morse wavelet analysis are plotted as images using the pcolor function in MATLAB to obtain their coefficient maps, adjusting the image size to 224 × 224 × 3 to be able to meet the small and large requirements of GoogLeNet for the input image.

## 4. GoogLeNet

**Figure 2:**
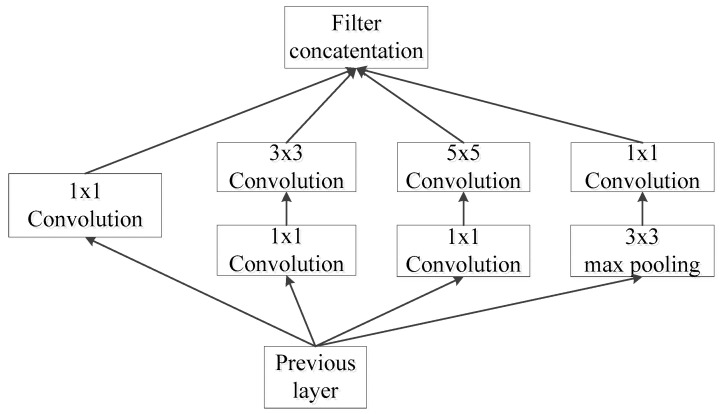
Structure diagram of Inception.

GoogLeNet [20] is a new deep learning structure proposed by Christian Szegedy in 2014; all the deep learning structures before this one obtain better training results by increasing the depth (number of layers) of the network, but the increase in the number of layers brings many negative effects [15], such as overfitting, gradient disappearance, and gradient explosion. The proposal of GoogLeNet, on the other hand, improves the training results from the perspective of increasing the network width of the convolutional network in extracting deep features; the Inception structure is introduced to fuse feature information at different scales, as shown in Figure 2. A 1 × 1 convolutional kernel is used for dimensionality reduction and mapping; two auxiliary classifiers are added; the fully connected layer is discarded and the average pooling layer is used, which greatly reduces the model parameters.

**Figure 3:**
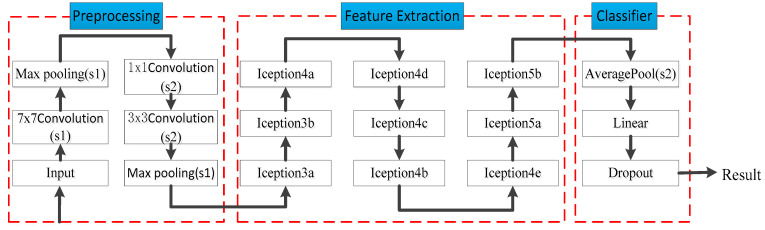
Network structure diagram of GoogLeNet.

The advantages of GoogLeNet compared to other deep learning structures are [15,21]: it can use computational resources more efficiently and extract more features with the same amount of computation, thus improving the training results. The network structure of GoogLeNet is shown in Figure 3. Among them, s1 and s2 represent the stride of the neural network module, s1 = 2 and s2 = 1. The whole network can be divided into three parts: pre-processing, feature extraction, and classifier. Among these, pre-processing is for adjusting the test data format to the format specified by Inception for input to extract features; feature extraction is composed of multiple Inceptions; the classifier consists of fully connected layer and dropout, where the activation function of Linear is chosen as the sigmoid function.

GoogLeNet is mostly used in the field of image processing and recognition. In this paper, the coefficient map obtained from the sound signal through continuous wavelet analysis is brought into GoogLeNet training to obtain the sound recognition model; the coefficient map obtained from the sound signal to be measured through continuous wavelet analysis is brought into the trained recognition model to realize the recognition and alarm of a coal mine gas or coal dust explosion.

## 5. Experimental Results and Analysis

### 5.1. Experimental Material

**Figure 4:**
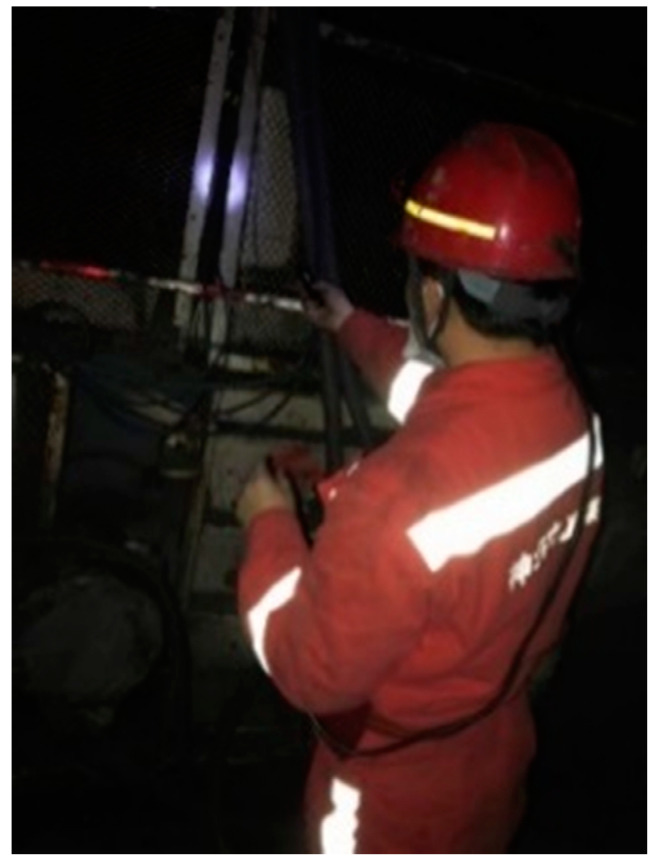
Sound collection scene.

In this paper, the experimental work of non-explosion sound data acquisition in the underground coal mine was carried out in the Shuangma coal mine of Shenhua Ningxia Coal Group, and the field acquisition work in the Shuangma coal mine is shown in Figure 4. The collection tools covered the key monitoring areas such as the comprehensive mining working face, comprehensive excavation working face, underground central distribution room, roadway, central water pump room, etc. The collected sounds include: the normal operation sound of equipment in the coal mining working face and excavation working face, rubber wheel car driving sound, water pump working sound, ventilator working sound, low-voltage feeder running sound, high-voltage distribution equipment running sound, primary distribution equipment running sound. The sound of a gas explosion and coal dust explosion was recorded by China Coal Industry Group Chongqing Research Institute Co. as shown in Table 1. The experimental algorithm verification was done on a DELL server with Inter i9-9980HK CPU@2.40 GHz, 32 Gb memory, and 64-bit OS using MATLAB2020a, and the sound editing was done using Goldwave software.

### 5.2. Wavelet Coefficient Map Extraction

**Figure 5:**
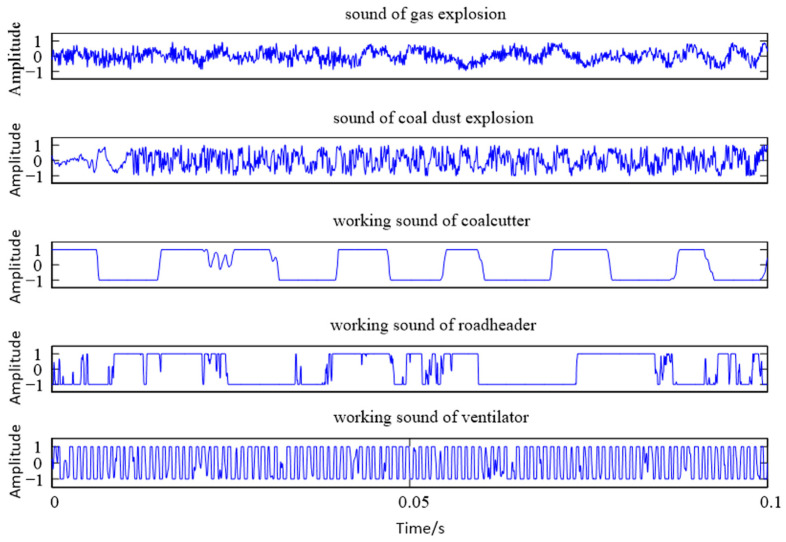
Time domain diagram of four kinds of sound: sound of gas explosion and coal dust explosion, working sound of shearer, roadheader, and ventilator.

In this section, continuous wavelet analysis is used as the theoretical basis to explore the feasibility and robustness of wavelet coefficient maps for characterizing sound signals. Due to the limitation of space, this paper takes gas explosion sound, coal dust explosion sound, coal mining machine working sound, roadheader working sound, and ventilator working sound as examples, and the time domain diagrams of the five sounds are shown in Figure 5. As can be seen from Figure 5, the time domain diagrams of the gas explosion sound and coal dust explosion sound have certain differences at the beginning of the sound stage, and the overall similarity is high; the time domain diagrams of the other three sound signals differ significantly, and the time domain characteristics alone cannot accurately determine the differences in their respective signals and do not have the conditions to be identified.

**Figure 6:**
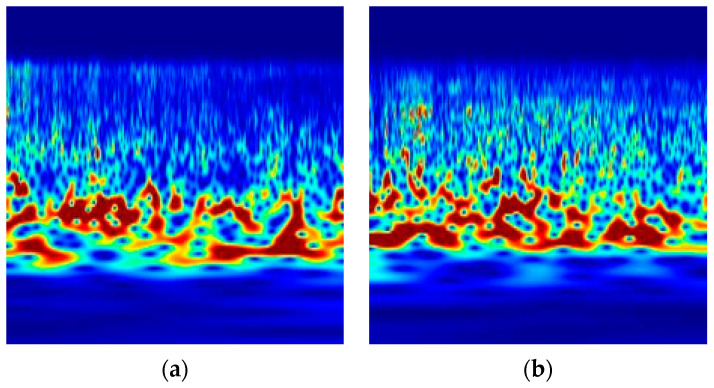

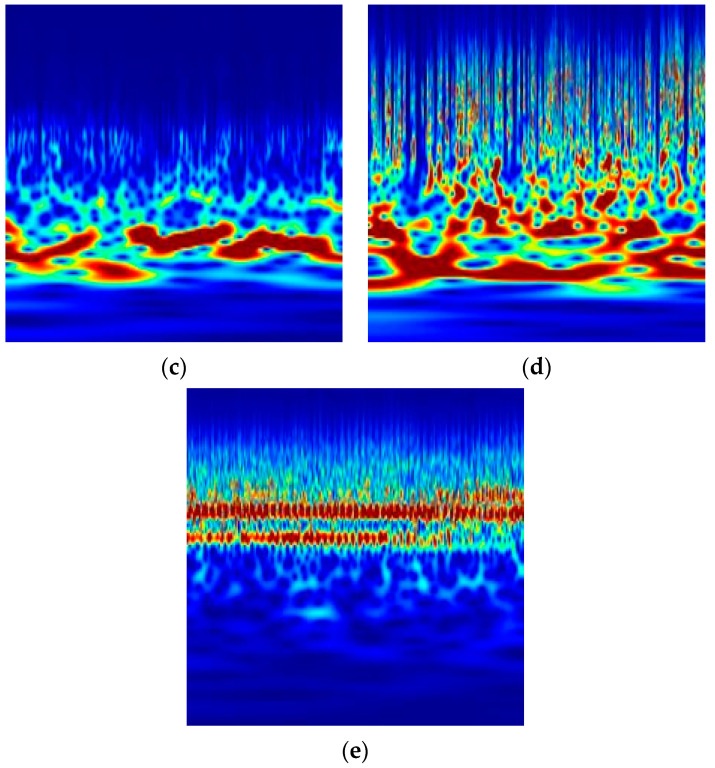
Scale factor diagram of different sounds: (**a**) sound of gas explosion, (**b**) sound of coal dust explosion, (**c**) working sound of coal mining machine, (**d**) working sound of roadheader, (**e**) working sound of ventilator.

To study the feasibility of the coefficient maps obtained by wavelet transform of five sound signals as feature extraction objects, in this paper, the coefficient maps obtained by wavelet transform of four sound signals of 0.5 s duration are shown in Figure 6. Figure 6a shows the wavelet coefficient map of the gas explosion sound, Figure 6b shows the wavelet coefficient map of the coal dust explosion sound, Figure 6c shows the wavelet coefficient map of the working sound of the coal mining machine, Figure 6d shows the wavelet coefficient map of the working sound of the roadheader, and Figure 6e shows the wavelet coefficient map of the working sound of the ventilator. In the figures, the horizontal axis represents time, and the vertical axis represents frequency.

From Figure 6, we can see that the wavelet coefficients of the gas explosion sound and the coal dust explosion sound have high similarity in distribution and are concentrated in the middle- and high-frequency part. The wavelet coefficients of the working sound of the coal mining machine are concentrated in the middle- and low-frequency part, while the wavelet coefficients of the working sound of the roadheader are scattered. The wavelet coefficients of the working sound of the ventilator are concentrated in the middle- and high-frequency part. The wavelet coefficients of the working sound of the ventilator are the most concentrated, followed by the working sound of the coal mining machine, followed by the sound of the gas explosion and the coal dust explosion. The worst is the working sound of the roadheader; the wavelet coefficient plots of the sound of the gas explosion and the coal dust explosion have high similarity and differ significantly from the wavelet coefficient distribution plots of the working sound of the coal mining machine, the working sound of the roadheader, and the working sound of the ventilator.

To objectively evaluate the feasibility of the wavelet coefficient map proposed in this paper, the mean value, entropy, standard deviation, and average gradient will be used as objective indicators to achieve the evaluation of the extracted wavelet coefficient map of the sound signals.

The larger the mean value of the image, the higher the image brightness, which can be calculated as

(4) $$M_{1}=\frac{1}{MN}\left[\overset{M}{\underset{a=1}{\sum }}\overset{N}{\underset{b=1}{\sum }}f\left(m,n\right)\right].$$

where *MN* is the size of the image and f(m,n) is the pixel value of the image at the coordinates (*m*,*n*).

The entropy of an image is a statistical form of a feature that reflects the average amount of information in the image, which can be calculated as

(5) $$H=-\overset{255}{\underset{i=0}{\sum }}p_{i}\cdot logp_{i}.$$

where $p_{i}$ is the probability that a certain grayscale appears in the image.

The standard deviation of the image indicates the degree of light and dark variation in the image, and the larger the standard deviation, the more obvious the light and dark variation in the image, which can be calculated as

(6) $$\delta =\sqrt{\frac{1}{MN}\overset{M}{\underset{a=1}{\sum }}\overset{N}{\underset{b=1}{\sum }}{\left(p\left(i,j\right)-u\right)}^{2}}.$$

where p(i,j) is the pixel value of the *i*th row and jth column, and u is the mean value.

The average gradient of the image means that there is a significant difference in grayscale near the boundary or both sides of the shadow line of the image; the rate of grayscale change and the magnitude of this rate of change can be used to indicate the image sharpness, which can be calculated as

(7) $$G=\frac{1}{MN}\overset{M}{\underset{a=1}{\sum }}\overset{N}{\underset{b=1}{\sum }}\sqrt{\frac{{\left(\frac{\partial f}{\partial x}\right)}^{2}+{\left(\frac{\partial f}{\partial y}\right)}^{2}}{2}}.$$

where $\frac{\partial f}{\partial x}$ is the gradient in the horizontal direction, $\frac{\partial f}{\partial y}$ is the gradient in the vertical direction.

The mean, entropy, standard deviation, and average gradient of the images were obtained by calculating the wavelet coefficient maps of the five sound signals, respectively, as shown in Table 2. From Table 2, we can see that the values of the four indicators of the wavelet coefficient maps of the gas explosion sound and the coal dust explosion sound are close in size, indicating that the brightness, average information, and clarity of the wavelet coefficient maps of the sound signals are similar, while the magnitudes of the wavelet coefficient diagrams of the working sound of the coal mining machine, the working sound of the roadheader, and the working sound of the ventilator are different.

Through the above analysis, it can be seen that the coefficient maps of the gas explosion sound and the coal dust explosion sound obtained by continuous wavelet analysis have similar values of parameter characteristics, which indicates that the similarity of the wavelet coefficient maps of the gas explosion sound and the coal dust explosion sound is high; the coefficient maps of the gas explosion sound and the coal dust explosion sound and other sounds in the coal mine underground obtained by continuous wavelet analysis have more obvious differences, which can effectively distinguish gas and coal dust explosion sounds and non-explosion sounds. Therefore, the wavelet coefficient maps obtained by continuous wavelet transform of the sound signals are feasible as image feature extraction objects of the GoogLeNet network and have high robustness.

### 5.3. Parameter Experiments

To select the appropriate parameters to achieve the optimization of the algorithm, this paper will conduct parameter experiments mainly including the dropout parameter of GoogLeNet and the initial learning rate. The dropout parameter is a proportion of neurons that are randomly ignored in the training process of the neural network model to achieve the joint adaptation between neurons and increase the generalization ability of the neural network. There must be an optimal solution for the initial learning rate, and the larger the value of the initial learning rate, the larger the value of the initial learning rate, leading to the occurrence of the non-convergence of the neural network recognition model, and the low initial learning rate will lead to the slow convergence speed or failure to learn. Therefore, this paper will select the appropriate dropout parameters and initial learning rate by experiment.

The dataset consists of 40 sets of gas explosion sounds and 172 sets of other sounds collected from underground coal mines, which are directly input to the GoogLeNet network. The training dataset is divided into training and test sets at a ratio of 9:1. The evaluation indexes for two parameters are recognition rate, accuracy rate, and recall rate. The formulas of precision rate and recall rate can be calculated as

(8) $$P=\frac{TP}{TP+FP}\times 100\%.$$

(9) $$R=\frac{TP}{TP+FN}\times 100\%.$$

where *P* is the precision rate, *R* is the recall rate, *TP* denotes the frequency of predicting positive class samples as positive class, *FN* denotes the frequency of predicting negative class samples as negative class, *FP* denotes the frequency of predicting negative class samples as positive class. In this paper, the positive class is coal mine gas and coal dust explosion, and all the remaining sounds are negative classes.

**Figure 7:**
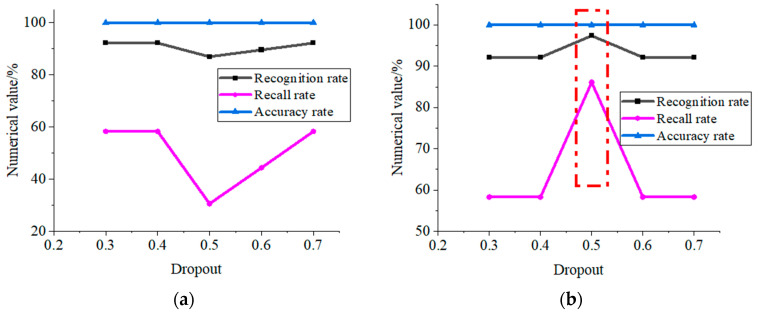
GoogLeNet recognition results with different parameters. (**a**) Learning rate is 0.0001; (**b**) learning rate is 0.001.

The value of the initial learning rate of GoogLeNet is generally set to 0.0001, and the value of the dropout parameter of GoogLeNet is generally set to 0.5. To choose more suitable parameters, this paper compares the experimental results with the value of the dropout parameter of 0.30.7 and the initial learning rate values of 0.0001 and 0.001, respectively. The recognition results are shown in Figure 7. Figure 7a shows the recognition results for different values of the dropout parameter when the initial learning rate is 0.0001, and Figure 7b shows the recognition results for different values of the dropout parameter when the initial learning rate is 0.001. From Figure 7, we can see that the recall rate of the proposed algorithm is 100% for different initial learning rates and different values of dropout parameter, which also shows that the algorithm of this paper can effectively distinguish gas and coal dust explosion sounds from non-explosion sounds; when the initial learning rate is 0.001, the overall recognition accuracy and recognition rate of the trained model are higher than that of the initial learning rate of 0.0001; when the initial learning rate is 0.001 and the dropout parameter value is 0.5, the recognition performance of the trained recognition model is optimal, with the recognition rate of 97.38%, accuracy rate of 86.1, and recall rate of 100%. The author also performed the same experimental analysis of the initial learning rate of 0.00001 and 0.01 with different dropout parameter values, and the experimental results show that with the initial learning rates of 0.00001 and 0.001, when the completed, the training model cannot distinguish between gas and coal dust explosion sounds and non-explosion sounds.

Combined with the above analysis, it can be seen that the coefficient map obtained by wavelet analysis proposed in this paper can effectively distinguish between gas and coal dust explosion sounds and non-explosion sounds and has high robustness. According to the test results of the experimental dataset, the parameter of GoogLeNet dropout is set to 0.5 and the initial learning rate is set to 0.001.

## 6. Experimental Results and Analysis

### 6.1. Experimental Results

To verify the superiority of the proposed method, the collected sound samples were edited by Goldwave software, and the edited sound signals were passed through the continuous wavelet transform to extract their wavelet coefficients. A total of 212 sets of data were involved in the test, including 40 sets of wavelet coefficients of gas and coal dust explosion sounds and 172 sets of wavelet coefficients of non-explosion sounds. The test datasets were directly input into the GoogLeNet network, and the ratio of test data to training data was set to 9:1 and 8:2, respectively. The test data and training data were brought into the three different neural networks of GoogLeNet, vgg, and Alexnet, and the results of the recognition experiments are shown in Table 3.

From the comparison results in Table 3, we can make some observations. (1) When the ratio of test data to training data is 9:1, the recognition rate of the gas and coal dust explosion sound recognition model proposed in this paper is 97.38%, which is 16.23% higher than that of VGG and 7.85% higher than that of Alexnet; the recall rate is 86.1%, which is 86.1% higher than that of VGG and 41.7% higher than that of Alexnet; the accuracy rate is 100%, which is 100% higher than VGG and tied with that of Alexnet (also 100%). (2) When the ratio of test data to training data is 8:2, the recognition rate, accuracy rate, and recall rate of the gas and coal dust explosion sound recognition model proposed in this paper are all 100%; compared with VGG, the recognition rate is 14.12% higher, the recall rate is 75% higher, and the accuracy rate is the same; compared with Alexnet, the recognition rate is 7.65% higher, the recall rate is 40.65% higher, and the accuracy rate is the same. It can be seen that the algorithm proposed in this paper still performs well in the case of a relative lack of training samples and can overcome the disadvantages of a large variety of sound samples and a small amount of data in underground coal mines. In terms of training time, Alexnet has the shortest training time, with 25 s and 45 s for 9:1 and 8:2, respectively, followed by the proposed algorithm with 124 s and 238 s, respectively, and VGG with 193 s and 385 s, respectively.

Combined with the above analysis, it can be seen that the proposed sound recognition model for gas and coal dust explosions has the best performance in terms of recognition rate, accuracy rate, and recall rate and can still identify and distinguish between coal mine gas and coal dust explosions and non-explosions more accurately in the absence of training samples. Although the training time is not the lowest, considering that the model training can be arranged during non-working time and the use of historical data for training the recognition classification model, this does not affect the real-time recognition classification work, which is also acceptable.

### 6.2. Experimental Analysis

The above experimental process and results show that the coefficient maps obtained by continuous wavelet analysis of gas and coal dust explosion sounds and other sounds in coal mines are significantly different, which can effectively distinguish between gas and coal dust explosion sounds and non-explosion sounds; the identification experiment results show that the proposed method has an excellent identification effect.

To avoid the pickups near the explosion source being damaged by high temperature and explosion shock waves, the mining pickups in this paper adopt a multi-point arrangement or use the existing mining camera’s pickups as a supplement. Although the explosion flame wave propagation speed is faster than the sound waves, the propagation distance of the explosion flame wave is smaller than the propagation distance of the explosion sound waves. A large number of mining pickups outside the vicinity of the explosion source will be saved for explosion sound recognition. We can determine the source of the explosion by monitoring and analyzing the sound characteristics of different monitoring locations, the sequence of monitoring gas and coal dust explosion sounds, and the sequence of damage to explosion-proof pickup equipment.

## 7. Conclusions

In this paper, a sound recognition method of coal mine gas and coal dust explosions based on GoogLeNet was proposed, using sound collected from the underground coal mine field as the experimental material. The following conclusions are drawn after several sets of experiments.

(1) The wavelet coefficient distribution of gas and coal dust sound signals obtained by continuous wavelet analysis is mainly concentrated in the middle and high frequencies, and the difference between the coefficient maps obtained by continuous wavelet analysis for gas explosion sounds and other sounds in coal mine shafts is more obvious. This can help effectively characterize gas and coal dust explosion sounds and non-explosion sounds and has strong robustness. It is feasible to use it as an image of GoogLeNet network feature extraction object.

(2) The parameters of dropout parameter and initial learning rate of GoogLeNet are determined through experiments. When the initial learning rate is 0.001, the overall recognition accuracy and recognition rate of the trained model are higher than that of the initial learning rate of 0.0001; when the initial learning rate is 0.001 and the value of the dropout parameter is 0.5, the recognition performance of the trained recognition model is optimal.

(3) Through comparison experiments with other classification models, it can be seen that the recognition rate, accuracy rate, and recall rate of the proposed recognition model are the best and can meet the needs of coal mine gas and coal dust explosion recognition. It can be applied to the recognition and alarm of abnormal sound in different application scenarios by modifying the training samples.

## Figures and Tables

**Table 1:** Sound material.

Sound Type	Total Duration/s	Number of Sound Clips	Data Volume/MB
Gas explosion sound	10	5	3
Coal dust explosion sound	10	5	3
Coal mine underground Non-explosion sound	8000	800	734

**Table 2:** Evaluation values of diagram.

Evaluation Indicators	Gas Explosion	Coal Dust Explosion	Coal Mining Machine	Roadheader	Ventilator
Mean	93.4	96.0	82.9	105.2	87.1
Entropy	6.2	6.2	5.7	7.1	6.2
Standard deviation	95.9	93.9	94.8	88.3	91.0
Mean gradient	11.4	13.8	6.2	21.3	10.7

**Table 3:** Recognition results of different classification models.

Percentage of Training	Model	Recognition Rate/%	Recall Rate/%	Accuracy Rate/%	Training Time/s
10%	GoogLeNet	97.38	86.1	100	124
	VGG	81.15	0	0	193
	Alexnet	89.53	44.4	100	25
20%	GoogLeNet	100	100	100	238
	VGG	85.88	25	100	385
	Alexnet	92.35	59.35	100	45

## Data Availability

Data sharing not applicable.
